# Multiparametric Mapping via Cardiovascular Magnetic Resonance in the Risk Stratification of Ventricular Arrhythmias and Sudden Cardiac Death

**DOI:** 10.3390/medicina60050691

**Published:** 2024-04-24

**Authors:** Maria Lo Monaco, Kamil Stankowski, Stefano Figliozzi, Flavia Nicoli, Vincenzo Scialò, Alessandro Gad, Costanza Lisi, Federico Marchini, Carlo Maria Dellino, Rocco Mollace, Federica Catapano, Giulio Giuseppe Stefanini, Lorenzo Monti, Gianluigi Condorelli, Erika Bertella, Marco Francone

**Affiliations:** 1 Humanitas Gavazzeni, 24125 Bergamo, Italy; 2 Department of Biomedical Sciences, Humanitas University, Via Rita Levi Montalcini, 4, 20090 Pieve Emanuele, Italy; 3 Humanitas Research Hospital IRCCS, Via Alessandro Manzoni, 56, 20089 Rozzano, Italy; 4 Centro Cardiologico Universitario, Azienda Ospedaliero-Universitaria Arcispedale S. Anna, 44124 Ferrara, Italy

**Keywords:** ventricular arrhythmias, sudden cardiac death, cardiovascular magnetic resonance, mapping

## Abstract

Risk stratification for malignant ventricular arrhythmias and sudden cardiac death is a daunting task for physicians in daily practice. Multiparametric mapping sequences obtained via cardiovascular magnetic resonance imaging can improve the risk stratification for malignant ventricular arrhythmias by unveiling the presence of pathophysiological pro-arrhythmogenic processes. However, their employment in clinical practice is still restricted. The present review explores the current evidence supporting the association between mapping abnormalities and the risk of ventricular arrhythmias in several cardiovascular diseases. The key message is that further clinical studies are needed to test the additional value of mapping techniques beyond conventional cardiovascular magnetic resonance imaging for selecting patients eligible for an implantable cardioverter defibrillator.

## 1. Introduction

Risk stratification for malignant ventricular arrhythmias and sudden cardiac death (SCD) is a daunting task for physicians in daily practice. Left ventricular (LV) ejection fraction is the main traditional imaging parameter used for SCD risk stratification in ischemic and non-ischemic heart diseases; however, it is not accurate in detecting myocardial tissue alterations, which could trigger ventricular arrhythmias [1,2]. For instance, myocardial fibrosis and edema modulate myocardial electrical properties and represent a potential substrate for malignant ventricular arrhythmias [3]. Conventional cardiovascular magnetic resonance (CMR) sequences can unveil focal myocardial edema and fibrosis through T2-weighted imaging and late gadolinium enhancement (LGE). LGE has been repeatedly associated with an increased risk of SCD in ischemic and non-ischemic cardiomyopathies [4,5,6,7] and has been implemented in daily practice for clinical decision-making [8]. The introduction of the novel sequences of parametric mapping has unveiled diffuse pathophysiological processes, including extensive myocardial inflammation and/or interstitial myocardial fibrosis, which could not be captured with conventional tissue characterization techniques [9]. These sequences provide the absolute quantification of the myocardial T1 and T2 relaxation values, potentially improving accuracy, reproducibility, sensitivity, and specificity for identifying underlying pathophysiological processes compared with conventional imaging [10].

T1 mapping reflects the longitudinal or spin lattice myocardial relaxation time, which is determined by how rapidly protons re-equilibrate their spins after being excited by a radiofrequency pulse. Modified look-locker inversion recovery (MOLLI) pulse sequences are among the most used CMR techniques for measuring T1 relaxation times over 17 successive heartbeats. A pixel-wise illustration of absolute T1 relaxation times is represented on a color map. Pre-contrast and post-contrast T1 mapping are used to derive the myocardial extracellular volume (ECV), given that gadolinium-based contrast agents are distributed throughout the extracellular space and shorten the T1 relaxation times of the myocardium proportionally to the local concentration of gadolinium. An estimation of the ECV can be obtained via the following formula:

$$ECV=(1-\;\mathrm{haematocrit}\;)\frac{\frac{1}{\;\mathrm{post}\;\mathrm{contrast}\;T1\;\mathrm{myo}\;}-\frac{1}{\mathrm{native}\;T1\;\mathrm{myo}\;}}{\frac{1}{\;\mathrm{post}\;\mathrm{contrast}\;T1\;\mathrm{blood}\;}-\frac{1}{\;\mathrm{native}\;T1\;\mathrm{blood}\;}}$$

T2 mapping reflects the transverse relaxation time, corresponding to the decoherence of transverse nuclear spin magnetization. It is assessed through pixel-wise fitting for a T2 decay curve of a series of T2-weighted sequences. Turbo-Spin-Echo sequences with varying echo times are typically used, but alternative sequences are commercially available [10].

**Figure 1:**
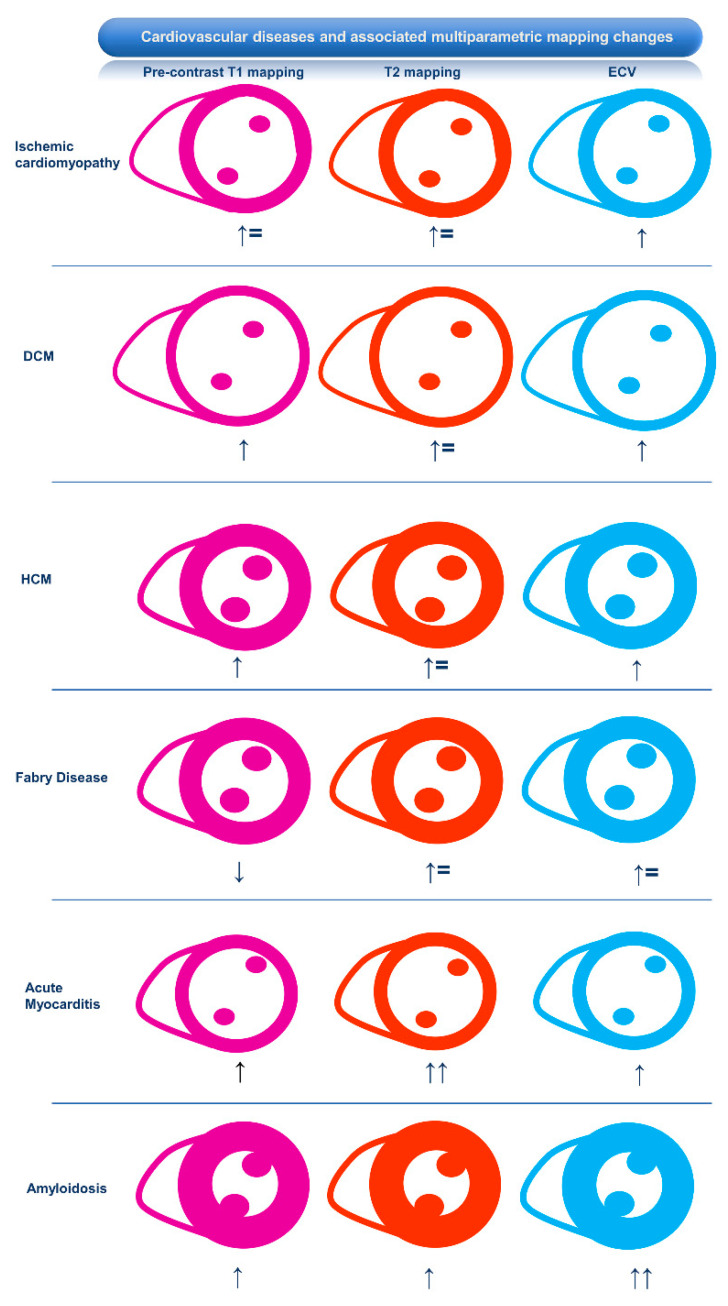
Cardiovascular diseases and associated multiparametric CMR mapping changes. From left to right, pre-contrast T1 mapping, T2 mapping, and ECV variations are shown. ↑: increased; ↑↑: markedly increased; ↑=: slightly increased or normal; ↓: reduced; DCM: dilated cardiomyopathy; HCM: hypertrophic cardiomyopathy.

T2 mapping values are increased because of edema associated with acute myocardial inflammation or necrosis. Pre-contrast T1 mapping values are reduced in the presence of sphingolipid storage. Pre-contrast T1 mapping and ECV values are increased in the case of acute inflammation or necrosis, replacement fibrosis, and diffuse fibrosis [11] (see Figure 1).

Cardiovascular diseases can predispose individuals to ventricular arrhythmias through several underlying structural mechanisms. An expansion of the myocardial extracellular space leads to mechanical and vasomotor dysfunction, key elements of electrical vulnerability. Increased automaticity can result from alterations to basic cellular ion exchange secondary to several myocardial pathologies. The latter can also represent electrical obstacles, paving the way for re-entry arrhythmias. Moreover, myocardial inflammation can alter cell action potentials, triggering abnormal impulse initiation [3,12].

By sensitively and accurately unveiling potentially arrhythmogenic tissue alterations, mapping sequences are promising features with which to improve SCD risk stratification. The present review explores the current evidence supporting the association between these mapping abnormalities and the risk of malignant ventricular arrhythmias/SCD in ischemic and non-ischemic cardiomyopathies (see Table 1).

## 2. Association of CMR Mapping Alterations and Ventricular Arrhythmias in Cardiovascular Diseases

### 2.1. Ischemic Heart Disease

Areas with previous myocardial infarction are characterized by increased ECV and native T1 values and normal T2 values. These mapping changes indicate the replacement of myocyte loss by scar, a potential substrate for ventricular arrhythmias [12]. In a cohort of consecutive patients (130 patients: 71 ischemic and 59 non–ischemic) undergoing CMR, pre-contrast T1 values were significantly higher in patients experiencing a study endpoint including appropriate implantable cardioverter–defibrillator (ICD) therapy or sustained ventricular tachycardia [2]. Indeed, a recent study showed that diffuse myocardial fibrosis quantified by ECV is associated with ventricular arrhythmias requiring ICD therapy in a dose–response fashion and provides superior discrimination compared with focal fibrosis identified via LGE [34]. 

In the context of an acute myocardial infarction, intramyocardial hemorrhage secondary to reperfusion damage leads to reduced T1 and T2 mapping values because of the paramagnetic effect of hemoglobin degradation products in the infarct core [10]. 

Native T1 mapping values are also reduced in lipomatous metaplasia within the area of myocardial infarction [13]. The presence of fat alters the electrical properties of the myocardium and might play a role in post-myocardial infarction arrhythmogenesis [35]. 

To the best of our knowledge, the role of T2 mapping as a marker of ventricular arrhythmias has not been explored. Overall, there is very limited evidence suggesting a potential role for multiparametric mapping in the identification of patients with ischemic heart disease and an increased risk of ventricular arrhythmias, but additional clinical studies may provide further clarification. 

### 2.2. Inflammatory Cardiomyopathy

Ventricular arrhythmias are common in inflammatory cardiomyopathy, and 20–40% of cases of SCD have been associated with myocardial damage secondary to myocardial inflammation [36]. Increased pre-contrast T1 mapping values or ECVs may be secondary to edema occurring in areas of active inflammation or irreversible fibrotic tissue alterations after the acute phase of the disease has resolved [37]. Increased T2 mapping values only reflect active inflammation and are not impacted by underlying fibrosis, providing better differentiation between the active and chronic phases of inflammatory diseases [38]. 

#### 2.2.1. Myocarditis

**Figure 2:**
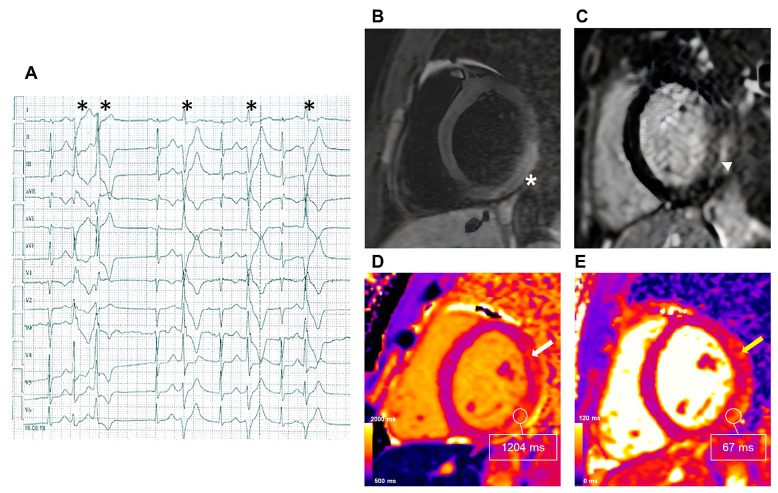
CMR findings in patients with acute myocarditis presenting with ventricular arrhythmias. A 42-year-old female was admitted to the emergency department for palpitations, pre-syncope, and chest pain two days before she performed an ECG Holter with evidence of frequent premature ventricular complexes and couplets, with an RBBB morphology and superior axis (**A**; asterisks); 1.5 T CMR was carried out three days later. (**B**) T2W-TSE image in the short-axis plane revealing the high signal intensity of the LV infero-lateral wall (asterisk). (**C**) T1W post-contrast delayed inversion recovery sequences demonstrating areas of enhancement of the subepicardial region of the myocardium with a normal subendocardial layer (short arrow). (**D**) Short-axis native T1 mapping with an average of 1110 ms, 1047 ms for the mid-septum, 1204 ms for the infero-lateral wall, and a reference value < 950 ms. (**E**) Short-axis T2 mapping revealing increased values with an average of 60 ms, 53 ms for the mid-septum, and 67 ms for the infero-lateral wall, with a reference value < 55 ms. The tissue alterations were more evident and extensive in mapping sequences than those shown by conventional sequences, also affecting the antero-lateral LV wall (white and yellow arrows in (**D**,**E**), respectively). CMR: cardiovascular magnetic resonance; LV: left ventricular; RBBB: right bundle branch block.

The proportion of SCDs attributed to myocarditis at autopsy varies by age, causing approximately 2% of infant (0–2 years), 5% of childhood (3–18 years), and less than 10% to 20% of young (19–44 years) SCDs [39,40,41]. Recent evidence about parametric mapping in myocarditis stems from studies regarding immune checkpoint inhibitor (ICI)-related myocarditis, a condition associated with the use of ICIs, drugs targeting the host immune regulatory pathways used in cancer therapy for an increasing number of malignancies, in some as a first-line therapy. ICI-related myocarditis is an uncommon immune-related adverse event but is associated with high reported mortality [42]. Thavendiranathan et al. demonstrated an independent association between higher T1 mapping values and cardiovascular events in a cohort of patients with ICI-related myocarditis. This association, however, could not be replicated for T2 mapping values [15]. In patients with clinically suspected acute myocarditis, an ECV ≥ 35% was found to be independently associated with a composite endpoint, including all-cause death, heart failure hospitalization, heart transplantation, documented sustained ventricular arrhythmia, and recurrent myocarditis [14]. Importantly, only the latter maintained a significant association with clinical outcomes in a multivariable model including age, LV ejection fraction, LGE, and increased ECV. An example of the role of mapping sequences in myocarditis is shown in Figure 2.

#### 2.2.2. Sarcoidosis

The incidence of SCD in cardiac sarcoidosis is exceptionally high, at up to 10.7% [43]. The presence of an increased T2 mapping signal has been associated with more frequent adverse cardiac events, including significant arrhythmias (both atrial and ventricular) and related symptoms (palpitations or near syncope) [17]. Crouser et al. have shown an association between T2 elevation and electrophysiologic study abnormalities (atrial arrhythmia, ventricular arrhythmia, atrioventricular block, or a QRS complex duration > 120 ms) [16]. In their population, the authors found that increased T2 mapping values in conjunction with LGE better predicted electrocardiographic abnormalities and arrhythmias compared with either parameter alone. 

#### 2.2.3. Connective Tissue Disorders

Autoimmune diseases affect the myocardium diffusely, and mapping sequences have shown increasing diagnostic values compared with LGE sequences [44]. However, a recent study of thirty-four patients with systemic sclerosis found no association between ventricular arrhythmias and CMR multiparametric mapping alterations in asymptomatic patients [45].

#### 2.2.4. Chagas Disease

The incidence of SCD is relevant in Chagas disease, and risk stratification is poor with conventional assessment. The chronic phase of Chagas myocarditis results in extensive myocardial fibrosis and LV aneurysms, predisposing the individual to ventricular arrhythmias. CMR is key to unveiling myocardial fibrosis, which is often transmural, resembling myocardial infarction, involving the LV apex and basal infero-lateral wall [46]; however, studies on mapping abnormalities are scarce. A study including 90 patients with Chagas disease demonstrated that remote native T1 values of greater than 1100 ms were predictive of the composite endpoint, including ICD implantation, heart transplantation, and death [19]. 

#### 2.2.5. Takotsubo Cardiomyopathy

Takotsubo cardiomyopathy is a reversible condition characterized by inflammation and edema, potentially associated with SCD [47,48]. Increased T2 mapping values typically normalize early after the acute phase, whereas increased T1 mapping values might persist for months after the acute phase, despite the normalization of the LV ejection fraction and chamber dimensions and a normal ECV [49,50,51,52,53,54]. To the best of our knowledge, no study has explored the association between mapping alterations and ventricular arrhythmias in this condition. 

Overall, further studies are necessary to corroborate a clinical role for multiparametric mapping in detecting patients with inflammatory heart diseases at increased risk of malignant ventricular arrhythmias. 

### 2.3. Hypertrophic Cardiomyopathy and Phenocopies

#### 2.3.1. Hypertrophic Cardiomyopathy

Predicting the risk of SCD in patients with hypertrophic cardiomyopathy (HCM) is crucial to selecting individuals who could benefit from prophylactic ICD implantation. The evaluation of LGE, especially if assessed quantitatively, has dramatically improved risk stratification as it is a high-risk feature of adverse outcomes [55,56,57] and has been consequently incorporated in currently recommended guideline algorithms. However, in addition to LGE, HCM is also typically characterized by diffuse myocardial fibrosis, which cannot be accurately distinguished via LGE, in contrast to T1 mapping and ECV. In a prospective study evaluating predictors of major adverse cardiovascular events in 203 HCM patients, it was found, via multivariate analysis, that native T1 was associated with adverse outcomes (HR 1.45; *p* < 0.001), even in a subgroup of patients judged as low-risk per European and American guidelines [20]. In a study of 73 patients with HCM [21], global ECV was the best parameter with which to identify patients with a risk of SCD ≥ 4% and patients with syncope or non-sustained ventricular tachycardia (NSVT) at the follow-up. Using a cut-off value of 34%, the global ECV had an area-under-the-curve (AUC) of 0.83 for identifying patients at a higher risk of SCD, which is significantly higher than that determined via LGE. Similarly, ECV performed better than LGE in identifying patients with syncope or NSVT, and the addition of ECV to the recommended SCD risk score provided the best discriminatory ability to identify patients who could benefit most from ICD implantation. Another study of 108 HCM patients [22] suggested that ECV was an independent predictor of SCD (HR 1.27, *p* < 0.001), and, compared with T1 mapping parameters, LGE, and conventional risk score stratification, it was the most potent predictor of SCD with good discriminatory ability (AUC 0.85). 

Post-contrast T1 values, an expression of interstitial myocardial fibrosis, were found to be associated with NSVT and aborted SCD, in a cohort of 100 patients with HCM [23]. While LGE presence did not differ between patients presenting with and without NSVT, patients with NSVT had significantly reduced values upon post-contrast T1 mapping. 

Higher values upon T2 mapping, potentially signaling edema due to ischemia or microvascular dysfunction, are commonly found both in the hypertrophied and non-hypertrophied segments in HCM patients compared with normal controls [58]. In a prospective study of almost 700 patients with HCM [24], during a median follow-up of 3 years, patients with LGE and higher T2 values had a higher risk of being at the composite endpoint of cardiovascular death and appropriate ICD shocks. Including T2 mapping significantly increased the predictive performance of established risk factors, including extensive LGE.

**Figure 3:**
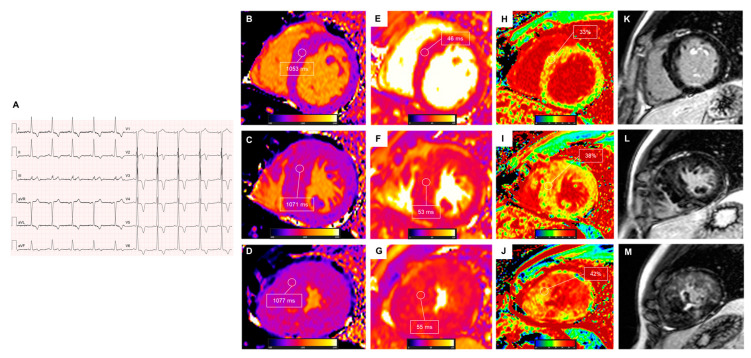
CMR findings in hypertrophic cardiomyopathy. A 31-year-old man with known apical hypertrophic cardiomyopathy was admitted for revaluation. The basal ECG is shown (**A**). Cardiovascular magnetic resonance showed elevated global T1 values (normal values < 1020 ms, (**B**–**D**)), T2 values at the upper limit of normality at the LV apex (55 ms, (**E**–**G**)), elevated global ECV values (normal values < 28%, (**H**–**J**)), and extensive LGE (28% of LV mass, (**K**–**M**)) involving the hypertrophied mid and apical segments. Multiparametric mapping abnormalities manifested a gradient from base to apex and demonstrated that interstitial fibrosis was also present in the basal LV segments, which presented normal wall thickness and no scar at LGE sequences. LGE: late gadolinium enhancement; LV: left ventricular; ECV: extracellular volume. Reproduced with permission from Stankowski et al. [20].

Nevertheless, further prospective work is needed to establish the role of myocardial mapping parameters as prognostic factors in HCM and to integrate that information into current clinical algorithms. At present, the quantitative evaluation of LGE among other clinical and imaging predictors remains crucial for risk stratification. Patients presenting with significantly elevated T1, T2, or ECV values and lacking conventional risk factors should probably be followed more closely as they might carry a higher risk of ventricular arrhythmias. A lower threshold for ICD implantation could be considered, while conclusive evidence is awaited. An example of multiparametric mapping in HCM is presented in Figure 3.

#### 2.3.2. Fabry Disease

Given that a reduction in native T1 mapping reflects globotriaosylceramide (Gb3) myocardial accumulation occurring before LV hypertrophy becomes manifest, CMR-based mapping allows an early diagnosis of Fabry disease (FD) cardiac involvement [59]. FD cardiomyopathy progression leads to LV hypertrophy, a “pseudo-normalization” of T1 mapping values, increased ECV, and eventually LGE in the infero-lateral LV wall [59,60,61,62]. Recently, Orsborne et al. developed a prognostic model for predicting adverse cardiac outcomes in this cohort of patients. In their study, T1 dispersion (the standard deviation of per voxel, a single sample or data point, myocardial T1 relaxation times) was an independent predictor of a composite clinical outcome, which included ventricular tachycardia, aborted SCD, and appropriate ICD therapy. The authors hypothesized that a wider distribution of myocardial T1 relaxation times (i.e., T1 dispersion) would better reflect glycosphingolipid accumulation and consequent fibrosis/inflammation [25]. 

#### 2.3.3. Amyloidosis

In patients with amyloidosis, the incidence of malignant ventricular arrhythmias is relatively low compared with that of other cardiac diseases [63]. Mapping alterations have been shown to be predictors of all-cause mortality or heart failure [64,65], but there is currently no evidence to suggest a role for these tissue alterations in ventricular arrhythmias. 

### 2.4. Dilated Cardiomyopathy 

T1 mapping techniques show potential in improving risk assessments for ventricular arrhythmias in patients with dilated cardiomyopathy (DCM) [27,66,67]. Interstitial fibrosis, characterized by intrinsic myocardial remodeling due to complex pathophysiological processes affecting the myocardium diffusely (not just focally), as shown by LGE, has been recently associated with life-threatening arrhythmias and all-cause mortality in DCM. A higher ECV has been independently associated with a composite endpoint of cardiovascular death, hospitalization for heart failure, and appropriate ICD discharge [27]. 

**Figure 4:**
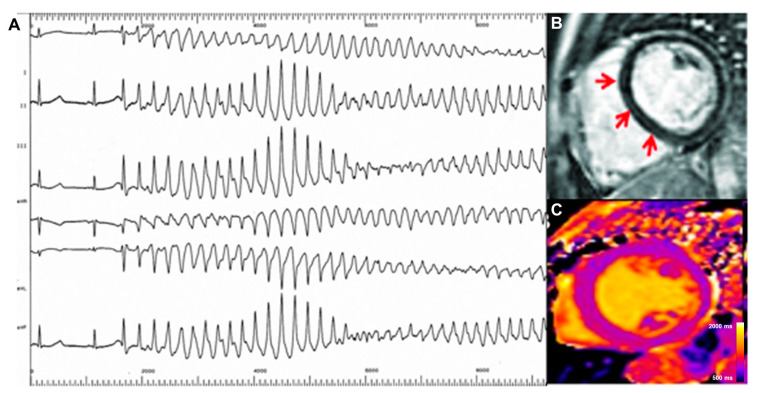
CMR findings in dilated cardiomyopathy presenting with ventricular arrhythmias. A 55-year-old man was referred to the cardiology department with a history of palpitations and progressive breathlessness. During an electrophysiologic study, a polymorphic ventricular tachycardia was induced (**A**). Cardiovascular magnetic resonance was performed and showed severe left ventricular dilatation with myocardial thinning and global hypokinesia. LGE sequences showed a mid-myocardial scar involving the septum (red arrows, (**B**)). Multiparametric mapping revealed markedly elevated global native T1 values (up to 1150 ms; normal range: 950–1050 ms), suggesting diffuse interstitial fibrosis (**C**). An implantable cardioverter defibrillator was implanted. LGE: late gadolinium enhancement.

In a recent investigation by Nakamori et al., DCM patients with a history of complex ventricular arrhythmias showed increased global native T1 values compared with age-matched DCM patients without any documented ventricular arrhythmia after adjusting for LV ejection fraction and LGE [26]. Pre-contrast T1 Z-scores and ECVs were independent predictors of arrhythmia-related events in a population of 225 patients with DCM [28]. In patients with DCM and without LGE, pre-contrast T1 and ECV values showed the best associations with a study endpoint, including heart failure, ventricular arrhythmias, and ICD or cardiac resynchronization therapy implantation, suggesting an added role for T1 mapping techniques on top of LGE conventional imaging [29]. Moreover, ECV might outperform LGE in the prediction of arrhythmias. In a population of patients with DCM, despite the similar distribution and extent of LGE between patients with and without ventricular arrhythmias, global and segmental ECVs were higher in the group of patients with arrhythmias (global ECV: 30.3 ± 4.2 vs. 27.9 ± 4.9; *p* < 0.02), in line with an independent association of global ECVs (HR 1.12, *p* < 0.02) with the arrhythmic burden [30]. T2 mapping values are altered in a subgroup of patients with DCM showing an underlying inflammatory background. However, there is no available evidence exploring the impact of these alterations on arrhythmic risk [66]. As noted above, the evidence supporting a role for T1 mapping techniques appears to be almost ready for primetime in daily practice. A case of DCM presenting with ventricular arrhythmias is shown in Figure 4.

### 2.5. Arrhythmogenic Right Ventricular Dysplasia/Cardiomyopathy

The annualized incidence rate of SCD in arrhythmogenic right ventricular dysplasia/cardiomyopathy (ARVD/C) is 0.06% [68]. Biventricular and left-dominant disease variants have been identified [69]. In the multiparametric tissue characterization of patients with ARVD/C, elevated pre-contrast T1 values are consistent with advanced fibrosis, and reduced values are consistent with fibrofatty infiltration. The thin right ventricular wall limits the feasibility of T1 mapping analysis [69]. Chun et al. retrospectively analyzed 60 patients with ARVD/C. Kaplan–Meier survival analysis revealed that heart failure-related events were more frequent in patients with increased values of pre-contrast T1 mapping and ECV. However, the authors found no association between mapping alterations and ventricular arrhythmias [31]. Further studies are awaited to explore the value of multiparametric mapping for the prediction of ventricular arrhythmias in arrhythmogenic cardiomyopathy. 

### 2.6. Mitral Valve Prolapse

A subgroup of patients with mitral valve prolapse (MVP) are exposed to an increased risk of SCD, the so-called “arrhythmic MVP”. Myocardial fibrosis, particularly in the sites most subject to the mechanical traction related to MVP mechanisms (i.e., papillary muscles and the LV posterior wall), is emerging as a detrimental player in this setting [70]. Notably, myocardial fibrosis depicted in pathological studies was “interstitial”, making it possible to be missed in conventional LGE, whereas T1 mapping techniques are potentially more accurate. Patients with MVP have shown increased pre-contrast T1 values in basal and mid-infero-lateral segments compared with other myocardial segments [71].

Accordingly, mapping techniques have been tested to identify patients at higher risk of arrhythmias. In an investigation including 23 patients with MVP underECG Holter monitoring, LV septal post-contrast T1 times were shorter in patients with complex ventricular arrhythmias compared with those without [33]. Accordingly, in another study including 30 patients with MVP, a basal infero-lateral ECV > 33.5% and LGE performed equally in identifying those with a history of aborted SCD. Among patients with available ECG Holter monitoring, ECV was more accurate than LGE in identifying those with complex ventricular arrhythmias, suggesting additional value beyond conventional tissue characterization in arrhythmic risk stratification [32]. These findings were not confirmed in a subsequent investigation including 42 patients with MVP, in which the ECV in the basal segments did not differ between patients with and without complex ventricular arrhythmias [72]. Similarly, no associations between T1 mapping values and complex ventricular arrhythmias were found in a study including 34 patients with MVP [73]. Thus, despite the potential theoretical advantages, mapping techniques have provided conflicting results on the association with ventricular arrhythmias in patients with MVP. Small sample sizes and methodological discrepancies in evaluating the arrhythmic outcome via T1 mapping may explain such inconsistencies.

## 3. Future Perspectives for CMR Mapping in Clinical Practice

The potential advantage of CMR mapping sequences in the clinical context of ventricular arrhythmias is the possibility of accurately identifying and quantifying arrhythmogenic pathological processes that escape conventional tissue characterization, ultimately with the goal of improving the risk stratification for SCD. As a result, the selection of patients undergoing primary prevention ICD implantation is expected to improve. Further development of more robust non-invasive cardiac imaging selection criteria could solidify the pathway that cardiologists and invasive cardiac electrophysiologists follow for the primary prevention of SCD [74]. ICD implantation impacts health system costs and quality of life, and may result in clinical complications [75]. Dedicated, large, multicenter studies comparing the potential benefits of mapping sequences to conventional tissue characterization (e.g., LV ejection fraction) are needed before the clinical implementation of these sequences in daily practice to select patients for ICD implantation and effectively prevent SCD. In this scenario, ECV might be preferred over absolute T1 mapping measurements given its better reproducibility and the lower influence of local variables on its values [76]. At present, however, multiparametric mapping is still underutilized due to its lower availability, the incomplete standardization of acquisition protocols, the need for local normal reference values (which hinders multi-center comparisons), the susceptibility to fast or irregular heart rates, and device-related artifacts [77].

Another potential application of CMR mapping sequences is in the guidance of invasive ablation procedures. Conventional LGE imaging is used for this purpose. It allows for the targeting of the arrhythmic substrate and evaluations of the location, depth, and possible gaps between radiofrequency lesions without ionizing radiation [78,79,80]. However, the lack of sensitivity and accuracy for subtle, diffuse pathological processes renders this approach prone to failure in some myocardial pathologies, particularly non-ischemic cardiomyopathies. In contrast, CMR mapping sequences might better delineate arrhythmogenic myocardial areas, reducing failure rates following LGE imaging alone. The latter relies on an arbitrary scale of the relative signal intensity difference detected between regions of dense scar and regions of user-defined “normal” tissue. Even in patients with ischemic heart disease, non-infarct regions seen as “normal” on contrast enhanced CMR imaging may contain diffuse interstitial fibrosis as a result of adverse remodeling and are potentially arrhythmogenic [2]. To our knowledge, CMR mapping sequences have yet to be tested for this potential clinical role. Moreover, parametric mapping, unlike LGE, would allow the repetition of the same sequence multiple times during an ablation procedure to evaluate lesion formation, potentially increasing the efficacy of ablation. Significant barriers towards widespread implementation are lack of availability and experience, the need for magnetic resonance-compatible interventional tools and suites, and the scarce clinical data available so far [78]. 

## 4. Conclusions

The present review highlights that the current evidence supporting the clinical use of mapping techniques to improve risk stratification for SCD, although promising, is unproven in most clinical contexts. Larger clinical studies are awaited to test the additional value of mapping techniques beyond conventional CMR imaging for selecting patients eligible for a primary prevention ICD in daily practice.

## Figures and Tables

**Table 1:** Evidence exploring the association between CMR multiparametric mapping and ventricular arrhythmias/sudden cardiac death in cardiovascular diseases.

Study First Author, Year	Type of Cardiomyopathy	Number of Patients	Type of Study	Mapping Parameter	Study Endpoint	Association of Mapping Parameter with the Study Endpoint
Chen, 2015 [2]	Ischemic cardiomyopathy	130	Prospective	10 ms increase of native T1 mapping	Appropriate ICD therapy or documented sustained VA	HR 1.1 (95% CI 1.0–1.2)
Olausson, 2023 [13]	Ischemic cardiomyopathy	215	Retrospective	5% increase in ECV	Time from ICD implantation to appropriate shock or anti-tachycardia pacing	HR 2.2 (95% CI 1.2–4.0)
Gräni, 2019 [14]	Myocarditis	179	Retrospective	ECV ≥ 35%	MACE (all-cause death, HF hospitalization, heart transplantation, documented sustained VA, and recurrent myocarditis)	HR 3.3 (95% CI 1.4–8.0)
Thavendiranathan, 2021 [15]	Myocarditis	136	Retrospective	Every 1-unit increase in T1 mapping z-score	MACE (cardiovascular death, cardiogenic shock, cardiac arrest, and complete heart block)	HR 1.4 (95% CI 1.1–1.8)
Crouser, 2014 [16]	Sarcoidosis	50	Retrospective	T2 mapping	Conduction system disease and cardiac arrhythmias (atrial arrhythmia, ventricular arrhythmia, atrioventricular block, or QRS complex duration > 120 ms)	T2 mapping significantly higher in patients with the study endpoint
Crouser, 2016 [17]	Sarcoidosis	8	Retrospective	T2 mapping > 70 ms	Reversible cardiac arrhythmias (atrial arrhythmia, ventricular arrhythmia, atrioventricular block, or QRS complex duration > 120 ms) after immune suppression therapy	T2 mapping significantly higher in patients with the study endpoint
Pinheiro, 2020 [18]	Chagas	62	Cross-sectional	T1 mapping > 1200 ms, ECV > 25%	NSVT	AUC 0.81 (95% CI 0.65–0.97) and 0.85 (95% CI 0.71–0.99)
Melo, 2023 [19]	Chagas	90	Prospective	Remote native T1 value > 1100 ms	ICD implantation, heart transplant, or death	HR 12 (95% CI 4.1–34.2)
Qin, 2021 [20]	HCM	203	Prospective	Native T1 mapping > 1300 ms	MACE (cardiac death, transplantation, HF admission, and ICD implantation)	HR 1.45 (95% CI 1.26–1.77)
Avanesov, 2017 [21]	HCM	73	Retrospective	Global ECV ≥ 35%	SCD, syncope, and NSVT	AUC 0.83 (95% CI 0.73–0.91)
Yu, 2023 [22]	HCM	108	Retrospective	Global ECV ≥ 35%	SCD	HR 1.27 (95% CI 1.10–1.47)
McLellan, 2016 [23]	HCM	100	Prospective	Post-contrast T1 mapping (median value: 422 ± 54 ms)	NSVT	Post-contrast T1 (*p* = 0.004)
Xu, 2023 [24]	HCM	674	Prospective	2 ms increase in T2 mapping	Cardiovascular death and appropriate ICD discharge	HR 1.43 (95% CI 1.18–1.72)
Orsborne, 2022 [25]	Fabry disease	200	Prospective	T1 dispersion	Adverse cardiac outcome (first hospitalization for HF, MI, coronary revascularization, VT sustained or nonsustained, new AF, bradyarrhythmia necessitating PM implantation, aborted SCD, appropriate ICD therapy, or cardiovascular death)	HR 1.012 (95% CI 1.002–1.021)
Nakamori, 2018 [26]	DCM	107	Retrospective	10 ms increase in T1 mapping	Complex VA	OR 1.14 (95% CI 1.03–1.25)
Barison, 2015 [27]	DCM	89	Retrospective	ECV > 29%	Cardiovascular death, hospitalization for HF and appropriate ICD intervention	*p* < 0.05
Cadour, 2023 [28]	DCM	225	Prospective	T1 mapping Z-score > 4.2, ECV > 30.5%	MACE (HF-related events and arrhythmia-related events)	HR 2.86 (95% CI 1.06–7.68) and HR 2.72 (95% CI 1.01–7.36)
Li, 2022 [29]	DCM	659	Retrospective	T1 mapping > 1000 ms, ECV > 30.5%	Cardiac-related death, heart transplantation, hospitalization for HF, VA, and ICD or CRT implantation	HR 1.13 (95% CI 1.10–1.36) and HR 1.32; (95% CI 1.12–1.53)
Rubiś, 2021 [30]	DCM	102	Prospective	ECV	Arrhythmic burden (ventricular tachycardia or a high burden of PVCs)	HR 1.12 (95% CI 1.00–1.25)
Chun, 2022 [31]	ARVD/C	60	Retrospective	T1 mapping, T2 mapping, and ECV	HF-related events (hospitalization, heart transplantation, and cardiac death) and ventricular tachycardia events	More HF-related events: higher native T1 (log-rank *p* = 0.002), T2 (log-rank *p* = 0.002), and ECV (log-rank *p* = 0.002)
Pavon, 2021 [32]	MVP	30	Retrospective	Synthetic ECV > 27%	Ventricular arrhythmic events (recent history of unexplained resuscitated OHCA and complex PVC)	AUC 0.83
Bui, 2017 [33]	MVP	41	Retrospective	Post-contrast T1 mapping	Complex VA	Reduced post-contrast T1 mapping in patients with complex VA

ARVD/C: arrhythmogenic right ventricular dysplasia/cardiomyopathy; AUC: area under the curve; CMR: cardiovascular magnetic resonance; HR: hazard ratio; OR: odds ratio; CI: confidence interval; MACE: major adverse cardiovascular events; HCM: hypertrophic cardiomyopathy; SCD: sudden cardiac death; NSVT: non-sustained ventricular tachycardia; ICD: implantable cardioverter-defibrillator; CRT: cardiac resynchronization therapy; PM: pacemaker; VA: ventricular arrhythmias; PVC: premature ventricular complex; HF: heart failure; MI: myocardial infarction; VT: ventricular tachycardia, AF: atrial fibrillation; MVP: mitral valve prolapse; VA: ventricular arrhythmias; OHCA: out-of-hospital cardiac arrest.

## Abbreviations

DCM	dilated cardiomyopathy
ECV	extracellular volume
FD	Fabry disease
HCM	hypertrophic cardiomyopathy
ICD	implantable cardioverter-defibrillator
ICI	immune check-point inhibitor
LGE	late gadolinium enhancement
LV	left ventricular
MVP	mitral valve prolapse
SCD	sudden cardiac death
